# Supplementary material for: The Relationship between Short- and Long-Term Memory Is Preserved across the Age Range
Source: Brain Sci. 2023 Jan 5;13(1):106. doi: 10.3390/brainsci13010106 (PMC9856639; doi:10.3390/brainsci13010106)
Supplement: Supplementary file 1 [file brainsci-13-00106-s001.zip › brainsci-1995529 - supplementary.pdf]

brain sciences

MDPI

Supplementary Information

Table S1. Supplementary statistics for Experiment 1, including main effects (a) and post-hoc comparisons (b).

a. Main effects

Identification accuracy

Effect	Statistic	p
Age group × Set size	*ATS*(1.90, ∞) = 1.04	0.35
Age group × Memory duration	*ATS*(1.99, ∞) = 0.05	0.95
Age group × Set size × Memory duration	*ATS*(1.89, ∞) = 1.43	0.24

Identification response time

Effect	Statistic	p
Age group × Set size	*ATS*(2.00, ∞) = 0.44	0.64
Age group × Memory duration	*ATS*(1.93, ∞) = 0.80	0.44
Age group × Set size × Memory duration	*ATS*(1.86, ∞) = 0.71	0.48

Localisation error

Effect	Statistic	p
Age group × Set size	*ATS*(1.96, ∞) = 1.02	0.36
Age group × Memory duration	*ATS*(1.96, ∞) = 1.93	0.14
Age group × Set size × Memory duration	*ATS*(1.98, ∞) = 2.02	0.13

b. Post-hoc comparisons

Identification accuracy

Effect	Statistic	z	p
Young vs Older	*U* = 7500.5	3.69	**<0 .001**
Middle-aged vs Older	*U* = 7208.5	2.66	**0.008**
Young vs Middle-aged	*U* = 6711.0	0.96	0.34
STM: 1-object vs 3 objects	*ATS*(1, ∞) = 33.76	n/a	**0.001**
LTM: 1-object vs 3 objects	*ATS*(1, ∞) = 0.01	n/a	0.91

Identification response time

Effect	Statistic	z	p
Young vs Older	*U* = 5265.00	−4.01	**< 0.001**
Middle-aged vs Older	*U* = 5576.00	−2.95	**0.003**
Young vs Middle-aged	*U* = 6201.00	−0.82	0.42
STM: 1-object vs 3 objects	*ATS*(1, ∞) = 141.64	n/a	**0.001**
LTM: 1-object vs 3 objects	*ATS*(1, ∞) = 1.70	n/a	0.19

Localisation error

Effect	Statistic		p
Young vs Older	U = 5434.0	-3.43	**< 0.001**
Middle-aged vs Older	U = 5557.0	-3.01	**0.003**
Young vs Middle-aged	U = 6303.0	-0.47	0.64
STM: 1-object vs 3 objects	$ATS(1, \infty)$ = 209.26	n/a	**0.001**
LTM: 1-object vs 3 objects	$ATS(1, \infty)$ = 40.40	n/a	**0.001**
